# Effect of ω-3 Polyunsaturated Fatty Acids-Derived Bioactive Lipids on Metabolic Disorders

**DOI:** 10.3389/fphys.2021.646491

**Published:** 2021-05-25

**Authors:** Jinjie Duan, Yayue Song, Xu Zhang, Chunjiong Wang

**Affiliations:** ^1^Department of Physiology and Pathophysiology, The Province and Ministry Co-Sponsored Collaborative Innovation Center for Medical Epigenetics, Tianjin Medical University, Tianjin, China; ^2^Tianjin Key Laboratory of Medical Epigenetics, Tianjin Medical University, Tianjin, China

**Keywords:** ω-3 PUFA, eicosanoids, metabolic disorders, diabetes, NAFLD, adipose tissue, atherosclerosis

## Abstract

Arachidonic acid (ARA) is an important ω-6 polyunsaturated fatty acid (PUFA), and docosahexaenoic acid (DHA), eicosapentaenoic acid (EPA) and n-3 docosapentaenoic acid (n-3 DPA) are three well-known ω-3 PUFAs. These fatty acids can be metabolized into a number of bioactive lipids. Eicosanoids derived from ARA have drawn great attention because of their important and complex biofunctions. Although EPA, DHA and n-3 DPA have also shown powerful biofunctions, we have fewer studies of metabolites derived from them than those from ARA. Recently, growing research has focused on the bioaction of ω-3 PUFA-derived metabolites, which indicates their great potential for treating metabolic disorders. Most of the functional studies of these bioactive lipids focused on their anti-inflammatory effects. However, several studies elucidated their direct effects on pancreatic β cells, hepatocytes, adipocytes, skeletal muscle cells, and endothelial cells. These researches revealed the importance of studying the functions of metabolites derived from ω-3 polyunsaturated fatty acids other than themselves. The current review summarizes research into the effects of ω-3 PUFA-derived oxylipins on metabolic disorders, including diabetes, non-alcoholic fatty liver disease, adipose tissue dysfunction, and atherosclerosis.

## Introduction

Polyunsaturated fatty acids (PUFAs) refer to fatty acids with two or more double bonds in their backbone. Arachidonic acid (ARA) is an important ω-6 PUFA, which can be metabolized from linoleic acid (Schmitz and Ecker, 2008). Docosahexaenoic acid (DHA), eicosapentaenoic acid (EPA) and n-3 docosapentaenoic acid (n-3 DPA) are three well-known ω-3 PUFAs and they can be derived from α-linolenic acid (ALA). The estimated conversion rate of ALA to EPA was 8–20% in human, while that to DHA was 0.5–9%, even lesser (Stark et al., 2008). Those PUFAs are precursors of a series of bioactive lipids metabolized by cyclooxygenase (COX), lipoxygenase (LOX), and cytochrome P450s (CYPs) and autoxidized non-enzymatically (Zhang et al., 2015).

Eicosanoids derived from ARA have drawn great attention because of their important and complex biofunctions. Many studies have examined the functions of ARA metabolites, including prostaglandins, thromboxanes, leukotrienes, lipoxins hydroxyeicosatetraenoic acids, and epoxyeicosatrienoic acid. These metabolites play vital roles in many physiological and pathophysiological processes. The effects of dietary supplement of ω-3 PUFAs are mediated not only by the precursor *per se* and their metabolites but also by competing the enzymes with ARA in the eicosanoid-producing process (Calder, 2020b). The effects of ARA and ARA-derived eicosanoids are well documented by several reviews (Sonnweber et al., 2018; Calder, 2020b). However, although ω-3 PUFAs also showed powerful biofunctions, we have fewer studies of their derived metabolites than those of ARA. Thus, we focused on the ω-3 PUFA derived bioactive lipids in the current review.

Metabolic disorders, such as obesity, diabetes, non-alcoholic fatty liver disease (NAFLD), and cardiovascular disease greatly threaten human health, and the prevalence of the diseases is increasing worldwide (Lavie et al., 2009; Younossi et al., 2016, 2018; Glovaci et al., 2019). In metabolic diseases, the profile of metabolites derived from ω-3 PUFAs is changed because of disturbed PUFA metabolism (Wang et al., 2017; Laguna-Fernandez et al., 2018; Garcia-Jaramillo et al., 2019). In the current review, we summarize the growing research into the effect of ω-3 PUFA-derived bioactive lipids on metabolic disorders, including diabetes, NAFLD, adipose tissue dysfunction and atherosclerosis.

## The Metabolic Pathways of ALA, EPA, DHA, and n-3 DPA

**FIGURE 1:**
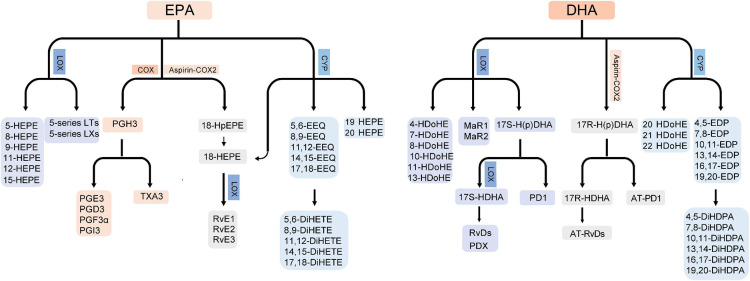
Biosynthesis of EPA/DHA-derived bioactive lipids. LT, leukotrienes; LX, lipoxins.

The metabolic pathways of ALA, EPA, DHA, and n-3 DPA were profoundly described by several reviews (Gabbs et al., 2015; Kuda, 2017; Drouin et al., 2019) and we briefly summarized as below:

ALA can be metabolized into hydroxy fatty acids by the COX and LOX pathway and epoxygenated fatty acids by the CYP pathway (Gabbs et al., 2015). In addition, ALA is the precursor of EPA, n-3 DPA and DHA. The rate limiting step is addition of a fourth double bond by Δ-6 desaturase. Next by elongation and desaturation, EPA is produced (Stark et al., 2008). EPA can be metabolized into 3-series prostaglandins and thromboxanes by the COX pathway; hydroxyeicosapentaenoic acids (HEPEs), E-series resolvins (RvE; RvE1-E3), 5-series leukotrienes and lipoxins by the LOX pathway; and epoxyeicosatetraenoic acids (EEQs) and dihydroxyeicosatetraenoic acids (diHETEs) by the CYP pathway (Zhang et al., 2015). Of note, 18-HEPE is derived from EPA by the CYP pathways or by aspirin-acetylated COX2 and then metabolized into RvEs by the LOX pathway (Figure 1; Gabbs et al., 2015).

**FIGURE 2:**
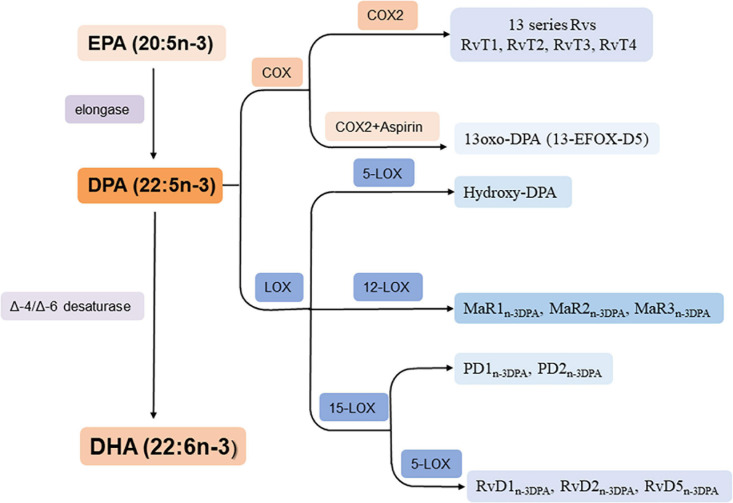
Biosynthesis of n-3 DPA-derived bioactive lipids. RvT1: 7,13R, 20-tri hydroxy-n-3 DPA; RvT2: 7,12,13R-tri hydroxy-n-3 DPA; RvT3: 7,8,13R-trihydroxy-n-3 DPA; RvT4: 7,13R-dihydroxy-n-3 DPA.

Docosahexaenoic acid can be metabolized into hydroxydocosahexaenoic acids (HDoHEs), D-series resolvins (RvD; RvD1-D6), maresins (MaR; maresin 1 and 2), protectins (PD; PD1 and PDX) by the LOX pathway and epoxydocosapentaenoic acids (EDPs) and dihydroxydocosapentaenoic acids (DiHDPAs) by the CYP pathway (Zhang et al., 2015). 17-hydroperoxydocosahexaenoic acid (17-H(p)DHA) is the precursor of DHA-derived specialized pro-resolving mediators. 17S-H(p)DHA can be metabolized from DHA by the LOX pathway and then metabolized into 17(S)-Hydroxy docosahexaenoic acid (17S-HDHA) and PD1. 17S-HDHA is further metabolized into RvDs and PDX. 17R-H(p)DHA is produced from DHA by aspirin-acetylated COX2 and then metabolized into 17R-HDHA and AT-PD1. 17R-HDHA can be further metabolized to AT-RvDs (Figure 1; Gabbs et al., 2015; Kuda, 2017).

n-3 DPA can be formed from EPA by elongase and converts to DHA by Δ6 orΔ4/-desaturase (Park et al., 2015; Drouin et al., 2019) thus it is an important intermediate in the conversion pathway of EPA and DHA (Figure 2). In addition, it can metabolized into PD_n–__3__DPA_ (PD1_n–__3__DPA_ and PD2_n–__3__DPA_), RvD_n–__3__DPA_ (RvD1_n–__3__DPA_, RvD2_n–__3__DPA_, and RvD5_n–__3__DPA_), MaR_n–__3__DPA_ (MaR1_n–__3__DPA_, MaR2_n–__3__DPA_, and MaR3_n–__3__DPA_) and hydroxy-DPA through LOX pathway; 13-series Rvs though COX pathway and 13-oxo derivatives by COX pathway when aspirin is existed (Figure 2; Drouin et al., 2019).

As ARA, ω-3 PUFA can also generate oxylipins non-enzymatically, which is mediated by uncontrolled oxidation (Galano et al., 2015; Hajeyah et al., 2020). ALA generates phytoprostanes, EPA generates F3-isoprostanes and DHA generates F4-neuroprostanes and neurofurans non-enzymatically (Galano et al., 2015).

In addition to ω-3 PUFA-derived oxylipins, conjugates of ω-3 PUFA with ethanolamine form acylethanolamides, which belong to fatty acid amides. Ethanolamine conjugates of DHA and EPA termed docosahexaenoyl ethanolamine (DHEA) and *N*-eicosapentaenoyl ethanolamine (EPEA), respectively (Meijerink et al., 2013). DHEA and/or EPEA can also be further metabolized by COX, LOX and CYP pathway (de Bus et al., 2019). DHEA and EPEA showed anti-inflammatory effects (de Bus et al., 2019), which indicates they may have bioactive effects on metabolic disorders. Besides, ω-3 PUFA intake was reported to reduced endocannabinoid levels in plasma and various tissues (Saleh-Ghadimi et al., 2020).

In the present review, we focus on the oxylipins enzymatically derived from ω-3 PUFA.

## The Identified Receptors of ω-3 PUFA-Derived Oxylipins

Identifying the receptors of these lipid mediators is vital to investigate their functions and the underlying mechanisms. Several studies have revealed that the effects of metabolites derived from ω-3 PUFA are mediated by G protein-coupled receptors (GPCRs) or nuclear receptors (Table 1). Krishnamoorthy et al. (2010) reported that RvD1 can directly bind to two GPCRs, ALX, and GPR32. ALX was first identified as an LXA4 receptor and GPR32 was considered an orphan receptor. The authors further revealed that RvD1-stimulated phagocytosis in macrophages was mediated by ALX and GPR32 (Krishnamoorthy et al., 2010). GPR18 is identified as a RvD2 receptor (Chiang et al., 2015). The protective effects of PDX on oxidative stress in vascular endothelial cells were mediated by GPR120, thus GPR120 may be a putative receptor of PDX (Hwang et al., 2019). MaR1 derived from DHA specifically binds to and activates human leucine-rich repeat containing G protein-coupled receptor 6 (LGR6) (Chiang et al., 2019). RvE1 binds to leukotriene B4 receptor 1 (BLT-1) and ERV-1 (also known as ChemR23) (Freire et al., 2017). 5-HEPE is an agonist of GPR119, a GPCR that regulates insulin secretion in pancreatic β cells (Kogure et al., 2011).

**TABLE 1:** The receptors of ω-3 PUFA-derived bioactive lipids.

**Metabolites**	**Precursors**	**Putative receptors**	
		**GPCR**	**NR**
RvD1	DHA	ALX	
		GPR32	
RvD2	DHA	GPR18	
MaR1	DHA	LGR6	RORα
PDX	DHA	GPR120	
RvE1	EPA	BLT-1	
		ERV-1	
5-HEPE	EPA	GPR119	PPARs
8-HEPE	EPA		PPARs
9-HEPE	EPA		PPARs
12-HEPE	EPA		PPARs
18-HEPE	EPA		PPARs

*GPCR, G protein-coupled receptor; NR, nuclear receptor.*

Peroxisome proliferator-activated receptors (PPARs) are nuclear receptors that can sense fatty acid and regulate lipid and glucose metabolism (Xu et al., 2018). The PPAR family includes three members, PPARα, PPARβ/δ, and PPARγ. HEPEs derived from EPA can activate PPARs (Yamada et al., 2014). 8-HEPE and 9-HEPE show higher ligand activities for PPARs than do 5-HEPE, 12-HEPE, 18-HEPE and EPA. Besides PPARs, MaR1 is an endogenous ligand of retinoic acid-related orphan receptor α (RORα) (Han et al., 2019). However, whether other ω-3 PUFA-derived metabolites are ligands of GPCRs or nuclear receptors is still unknown.

## Effect of ω-3 PUFA-Derived Oxylipins on Diabetes

Type 1 diabetes is described as immune-mediated destruction of pancreatic β cells, and the characteristics of type 2 diabetes are insulin resistance and progressive β-cell failure (Yang et al., 2018). Diabetes is a major metabolic disorder with high prevalence and is a risk factor for relevant public health issues such as cardiovascular disease, retinopathy, microangiopathy, and impaired wound healing (Yang et al., 2018).

EPA and DHA have shown beneficial effects for both type 1 and type 2 diabetes in rodents (Krishna Mohan and Das, 2001; Suresh and Das, 2003; Bi et al., 2017; Lepretti et al., 2018) and there is increasing evidence that the metabolites of EPA and DHA regulate these procedures. However, clinical trials showed conflicting results of dietary supplement of EPA/DHA on metabolic parameters in diabetic patients. A 6-month EPA treatment decreased postprandial glucose level of newly diagnosed impaired glucose metabolism patients (Sawada et al., 2016). Another clinical research also revealed the beneficial effects of ω-3 PUFA supplement on metabolic parameters including glucose and glycosylated hemoglobin in type 2 diabetic patients (Jacobo-Cejudo et al., 2017). However, several clinical studies revealed neutral effects of ω-3 PUFAs on metabolic profiles in type 2 diabetic patients (Wong et al., 2010; Poreba et al., 2017). The disagreement of these studies may be related with different sample sizes, baseline characteristics of patients, different doses and purities of these fatty acid, different time courses of the treatments and different basic medicine of these patients. Moreover, Poreba et al. (2017) also demonstrated high-dose ω-3 PUFAs did not increase RvD1 level in patients with atherosclerosis and type 2 diabetes and this is an important clue that the production of bioactive metabolites of ω-3 PUFAs is related to their therapeutic effects (Poreba et al., 2017). Thus, to study the effects and mechanism of ω-3 PUFA-derived metabolites is important to develop new strategies to confront diabetes.

Recently, the bioactive lipids derived from EPA or DHA, including RvD1, RvD2, PDX, RvE1, and 5-HEPE, were reported to affect insulin resistance or pancreatic β-cell function (Table 2). Moreover, ω-3 PUFA metabolites can be involved in diabetic complications, including impaired wound healing and diabetic retinopathy (Table 2).

**TABLE 2:** The functions of ω-3 PUFA-derived bioactive lipids on metabolic disorders.

**Metabolites**	**Function**	**Diseases**	***In vivo***	***In vitro***	**References**
RvD1	– Insulin resistance; – Adipose tissue Inflammation	Type 2 diabetes	√	√	Hellmann et al., 2011; Bathina et al., 2020
	– Oxidative stress – Inflammation	Type 1 diabetes	√		Bathina and Das, 2021
	+ Healing of diabetic wounds	Diabetic complications	√		Bathina and Das, 2021
	– Pro-angiogenic potential of retinal photoreceptors	Diabetic complications		√	Maisto et al., 2020
	– NASH	NASH	√	√	Rius et al., 2014; Li et al., 2020
	– Macrophage inflammation	Obesity		√	Titos et al., 2011
	– Advanced atherosclerosis	Atherosclerosis	√		Fredman et al., 2016
17-HDHA, RvD1 precursor	– Adipose tissue Inflammation	Obesity; Type 2 diabetes	√		Neuhofer et al., 2013
	– NAFLD; – Liver inflammation	NAFLD	√		Rodriguez-Echevarria et al., 2018
RvD2	– Adiposity; + Glucose tolerance	Obesity	√		Pascoal et al., 2017
Protectin DX	– Insulin resistance; + skeletal muscle IL-6 secretion	Type 2 diabetes	√	√	White et al., 2014
	– Skeletal muscle cell Insulin resistance	Type 2 diabetes	√	√	Jung et al., 2017
	– Hepatocyte insulin resistance; – Fetuin-A and selenoprotein	Type 2 diabetes		√	Jung et al., 2019
	– Adipocyte Inflammation; – Adipocyte Insulin resistance	Insulin resistance		√	Jung et al., 2018a
	– Hepatic steatosis	NAFLD	√	√	Jung et al., 2018c
MaR1	– TNFα induced lipolysis	Obesity		√	Laiglesia et al., 2018a
	– Insulin resistance; – Adipose tissue Inflammation; + Adiponectin secretion	Obesity; Type 2 diabetes	√	√	Martinez-Fernandez et al., 2017, 2020
	– Hepatic steatosis;	NAFLD	√	√	Rius et al., 2017; Jung et al., 2018b; Laiglesia et al., 2018b
	+ M2 polarity of liver macrophages	NASH	√	√	Han et al., 2019
MaR1 + RvD2	– Atherosclerosis; – Macrophage inflammatory	Atherosclerosis	√	√	Viola et al., 2016
PD1	+ Adiponectin secretion	Obesity		√	Gonzalez-Periz et al., 2009
19,20-DiHDPA	+ Diabetic retinopathy	Diabetic complications	√	√	Hu et al., 2017
19,20-EDP	+ Autophagy (hepatocyte); – Insulin resistance (adipocyte)	NAFLD; Obesity		√	Lopez-Vicario et al., 2015
RvE1	– Hepatic steatosis – Liver inflammation	NAFLD	√		Gonzalez-Periz et al., 2009
	– Atherosclerosis	Atherosclerosis		√	Salic et al., 2016
RvE1 (RvE1 receptor overexpression)	– Insulin resistance; – Inflammation	Obesity; Type 2 diabetes	√		Sima et al., 2017; Pal et al., 2020
18-HEPE/Resolvin E1 (RvE1 receptor deletion)	– Macrophage oxLDL uptake; – Atherosclerosis	Atherosclerosis	√	√	Laguna-Fernandez et al., 2018
18-HEPE	– NAFLD; – Liver inflammation	NAFLD	√		Rodriguez-Echevarria et al., 2018
	– Endothelial activation	Atherosclerosis		√	Liu et al., 2018
8-HEPE	– Dyslipidemia – Liver steatosis	NAFLD	√		Saito et al., 2020
17,18-EEQ	– Insulin resistance (adipocyte)	Obesity		√	Lopez-Vicario et al., 2015
	– Endothelial activation	Atherosclerosis		√	Liu et al., 2018
17,18-EEQ 9-HEPE 5-HEPE	– Liver steatosis; – Adipose tissue inflammation – Macrophage inflammation	NAFLD	√	√	Wang et al., 2017
5-HEPE	+ T-reg in adipose tissue	Obesity		√	Onodera et al., 2017
	+ Insulin secretion	Diabetes		√	Kogure et al., 2011
12-HEPE	+ Cold adaptation; +Glucose uptake (adipocyte and skeletal muscle)	Diabetes	√	√	Leiria et al., 2019
RvD5_n–__3__DPA_	– Leukocyte and platelet activation – Aortic lesions	Atherosclerosis	√	√	Colas et al., 2018
13-oxo-OTA	+ Glucose uptake (adipocyte)	Diabetes		√	Takahashi et al., 2015

*–, inhibit; +, promote; Ref., reference.*

### Effect of DHA-Derived Oxylipins on Diabetes

The levels of RvD1 and 17-HDHA were decreased in adipose tissue of genetic as well as diet-induced obese mice (Neuhofer et al., 2013). 17-HDHA treatment was further found to improve adipose tissue inflammation and insulin sensitivity in high-fat-diet (HFD)-fed mice (Neuhofer et al., 2013). Also, RvD1 has beneficial effects on insulin resistance. Hellmann et al. (2011) demonstrated that RvD1 improved glucose tolerance and increased insulin-stimulated pAkt level in liver, adipose tissue and skeletal muscle in db/db mice. The authors further found that RvD1 increased the ratio of M2 and M1 adipose-tissue macrophages (Bathina et al., 2020) and ameliorated adipose tissue inflammation (Hellmann et al., 2011). RvD1 was also reported to improve insulin resistance through the PI3K-Akt-mTOR axis in brain tissue (Bathina et al., 2020). *In vitro* study also indicated that RvD1 could attenuate interferon γ (IFN-γ)/lipopolysaccharide-induced pro-inflammatory cytokine expression in macrophages (Titos et al., 2011). Collectively, RvD1 improves insulin sensitivity by inhibiting tissue inflammation. Moreover, RvD1 ameliorated streptozotocin induced type1 diabetes in mice (Bathina and Das, 2021). In addition, local RvD1 delivery can accelerate wound closure in diabetic mice by stimulating macrophage phagocytosis to enhance clearance of apoptotic cells (Tang et al., 2013). *In vitro* study demonstrated RvD1 reduced the pro-angiogenic potential of retinal photoreceptors treated by high glucose by increasing anti-angiogenic miRNAs and decreasing VEGF content in exosomes (Maisto et al., 2020).

PDX-treated mice showed protection from lipid-induced insulin resistance. Along with this effect, PDX inhibited lipid-induced secretion of C-C motif chemokine ligand (CCL) 2, CCL5, tumor necrosis factor α (TNF-α), IFN-γ, interleukin 1β (IL-1β), IL-2, and IL-17. However, IL-6 level was significantly increased with PDX treatment, which was from skeletal muscle and suppressed gluconeogenic gene expression in liver (White et al., 2014). In addition, PDX can activate AMPK independent of IL-6 (White et al., 2014). Consistent with this finding, PDX improved HFD-induced insulin resistance in mouse skeletal muscle and palmitate-induced insulin resistance in skeletal muscle cells by activating AMPK and increasing PPARα expression (Jung et al., 2017). In hepatocyte, PDX ameliorated palmitate-induced insulin resistance by downregulating the expression of fetuin-A and selenoprotein P (Jung et al., 2019). Fetuin-A and selenoprotein P were hepatokines and their levels were increased in the plasma of obesity patients (Jung et al., 2019). PDX also improved lipopolysaccharide-induced insulin resistance in adipocytes (Jung et al., 2018a).

MaR1 treatment ameliorated insulin resistance in db/db mice and HFD-fed mice by suppressing inflammation and improving insulin sensitivity in adipose tissue (Martinez-Fernandez et al., 2017). The effects of MaR1 on insulin sensitivity were also confirmed in human adipocytes which were mediated by improving Akt activation (Martinez-Fernandez et al., 2020).

Soluble epoxide hydrolase (sEH) is a member of the epoxide hydrolase family in the CYP pathway (He et al., 2016). It hydrolyses EDPs into DiHDPAs. sEH expression and activity was found increased in retinas of diabetic mice, and the level of its product 19,20-DiHDPA was elevated in eyes. However, levels of other sEH substrates and products were comparable between control and diabetic mice. 19,20-DiHDPA was further found to increase endothelial cell permeability and induce the migration of pericytes into the extravascular space (Hu et al., 2017). Of note, the expression of sEH was increased in retinas of patients with non-proliferative diabetic retinopathy as compared with non-diabetic individuals (Hu et al., 2017), so sEH has potential as a therapeutic target of diabetic retinopathy.

### Effect of EPA-Derived Oxylipins on Diabetes

BLT-1 and ERV-1 are two receptors for RvE1 (Freire et al., 2017). In type 2 diabetic patients’ neutrophils, ERV-1 expression was significantly upregulated and BLT-1 expression was decreased. In addition, the serum level of RvE1 was decreased in type 2 diabetic patients versus healthy controls. RvE1 was further found to facilitate neutrophil phagocytosis from healthy individuals, and a higher dose was needed to achieve a similar response in neutrophils of diabetic patients (Freire et al., 2017). These data indicate that repressed RvE1 signaling is involved in neutrophil phagocytosis dysfunction in type 2 diabetes. In addition, overexpression of the RvE1 receptor ERV-1 in myeloid cells attenuated diet-induced obesity, hepatic steatosis and glucose intolerance in mice. A mechanism study revealed that ERV-1 overexpression maintained peripheral blood monocyte and adipose-tissue macrophage skewing to an M2 phenotype in mice with an HFD (Sima et al., 2017). Besides, RvE1 was reported to improve hyperinsulinemia and hyperglycemia in HFD fed mice by activating ERV-1. The authors further demonstrated genetic diversity and variability defined the therapeutic effects of RvE1 by using the diversity outbred mice. This research highlights the genetic variants in the RvE1 response need to be considered when exploring the therapeutic effects of EPA clinically (Pal et al., 2020).

Eicosapentaenoic acid could increase glucose-stimulated insulin secretion from ob/ob mice (Neuman et al., 2017) 5-HEPE derived from EPA could increase glucose-stimulated insulin secretion in MIN6 cells by activating the GPR119/cAMP pathway (Kogure et al., 2011). Thus, the effect of EPA on insulin secretion may be mediated by its metabolites, which needs further investigation.

## Effect of ω-3 PUFA-Derived Oxylipins on Non-alcoholic Fatty Liver Disease

Non-alcoholic fatty liver disease is defined as the accumulation of excess fat in the liver in the absence of excessive alcohol drinking and any secondary cause and thus a hepatic manifestation of metabolic syndrome (Ahmed, 2015). In NAFLD, simple steatosis can progress into non-alcoholic steatohepatitis (NASH), estimated to be the major reason for liver transplantation in the United States by 2020 (Diehl and Day, 2017). EPA and DHA showed protective effects on NAFLD (Scorletti and Byrne, 2018; Yan et al., 2018; Jordao Candido et al., 2019). Moreover, to better understand the underlying mechanisms, increasing studies have focused on the functions of their derived metabolites in NAFLD.

### Effect of ω-3 PUFA-Derived Oxylipins on Hepatic Steatosis

Hepatic steatosis is considered the first hit in the current “multiple-hit” theory proposed for the pathogenesis of NAFLD (Ahmed, 2015). PDX, MaR1, 19,20-EDP, and 17-HDHA derived from DHA and 17,18-EEQ, 18-HEPE, and RvE1 derived from EPA showed potential to ameliorate hepatic steatosis (Table 2).

PDX and MaR1 suppress palmitate-induced lipid accumulation in hepatocytes by attenuating endoplasmic reticulum stress (Rius et al., 2017; Jung et al., 2018b, c). For the mechanism, MaR1 activated AMPK and then induced sarcoendoplasmic reticulum Ca^2+^-ATPase 2b expression, which alleviated the palmitate-induced endoplasmic reticulum stress (Jung et al., 2018b). Consistent with the *in vitro* study, in HFD-fed mice and ob/ob mice, MaR1 alleviated hepatic steatosis (Jung et al., 2018b; Laiglesia et al., 2018b). 18-HEPE and 17-HDHA could improve HFD-induced hepatic steatosis. Also, 18-HEPE and 17-HDHA increased adiponectin level in HFD mouse (Rodriguez-Echevarria et al., 2018). However, whether the beneficial effects of 18-HEPE and 17-HDHA depend on adiponectin need further studies. 18-HEPE is the precursor of RvE1. Moreover, intraperitoneal injection of RvE1 significantly ameliorated the hepatic steatosis and inflammation of ob/ob mice (Gonzalez-Periz et al., 2009). Our recent study found that 17,18-EEQ, 5-HEPE and 9-HEPE derived from EPA ameliorated short-term HFD-induced liver steatosis by attenuating adipose tissue inflammation. In the study, we also found the anti-inflammatory effect of HEPEs and EEQs was more pronounced than the same dose of EPA (1 μM), although EPA at 50 μM showed a significant anti-inflammatory effects (Wang et al., 2017). In addition, 8-HEPE improved dyslipidemia and liver steatosis in low-density lipoprotein (LDL) receptor deficient mice fed with high cholesterol diet (Saito et al., 2020).

sEH can decrease EEQ and EDP level by hydrolyzing them into less active diols (He et al., 2016). Inhibition of sEH reinforced the protective role of *fat-1* transgenic mice in HFD-induced liver inflammation and steatosis by increasing 17,18-EEQ and 19,20-EDP production (Lopez-Vicario et al., 2015). For the mechanism, 19,20-EDP and 17,18-EEQ ameliorated insulin signaling in palmitate-treated adipocytes; 19,20-EDP restored autophagy in palmitate-treated hepatocytes (Lopez-Vicario et al., 2015).

### Effect of ω-3 PUFA-Derived Derived Oxylipins on Non-alcoholic Steatohepatitis

Non-alcoholic steatohepatitis is characterized by liver steatosis, inflammation, hepatocellular injury and different degrees of fibrosis and is the progressive form of NAFLD (Schuster et al., 2018). A recent study found RvD1 treatment mitigated lipid accumulation, inflammation and hepatic fibrosis in MCD-diet induced NASH mice. For the mechanism, RvD1 suppressed oxidative stress by activating nuclear factor E2-related factor 2 and ameliorated inflammation by inhibiting NF-κB and MAPK signaling pathways (Li et al., 2020). In addition, RvD1 had additional protective effects on calorie restrictive-improved NASH, as evidenced by decreased macrophage infiltration with decreased expression of M1 macrophage markers and increased expression of M2 macrophage markers (Rius et al., 2014). Also, Han et al. (2019) demonstrated that MaR1 derived from DHA increased the M2 polarity of liver macrophages and then ameliorated NASH by activating RORα. RORα, as a nuclear receptor, in turn increased MaR1 production by transcriptional induction of 12-lipoxygenase expression (Han et al., 2019). These studies suggest that these specialized pro-resolving lipid mediators derived from ω-3 PUFAs have therapeutic potential for NASH by promoting M2 polarization of liver macrophages.

## Effect of ω-3 PUFA-Derived Oxylipins on Adipose Tissue Function

Depending on the adipocyte, adipose tissue can be divided into white and brown adipose tissue. Also, inducible cells within white adipose tissue, called “beige” adipocytes, can generate heat under cold exposure (Rosen and Spiegelman, 2014; Ye et al., 2020). Adipose tissue functions, including adipose tissue inflammation, lipolysis, adipogenesis, endocrine function, and browning, are closely related to obesity-related diseases. The studies of the effects of ω-3 PUFA derivatives on adipose tissue function mainly focused on the immune response of adipose tissue. Their influence on macrophage function contributing to adipose tissue inflammation was discussed in the previous section (Table 2). In addition, Onodera et al. (2017) demonstrated that EPA increased the number and proportion of T regulatory cells in epididymal adipose tissue of db/db mice. This result was mediated by 5-HEPE, which is derived from EPA by 5-LOX (Onodera et al., 2017).

In addition to the immune response, other adipose tissue functions are regulated by ω-3 PUFA-derived bioactive metabolites.

### Effect of ω-3 PUFA-Derived Oxylipins on Lipogenesis and Lipolysis

The imbalance of lipogenesis and lipolysis of adipose tissue can increase the risk of obesity-induced disease (Lafontan, 2014). MaR1 inhibited TNF-α-induced lipolysis in 3T3-L1 adipocytes (Laiglesia et al., 2018a). Increased adipocyte lipolysis may increase plasma free fatty acid level and lead to insulin resistance and fatty liver disease (Matsuzaka and Shimano, 2011).

Nevertheless, PDX treatment inhibited lipid accumulation in 3T3-L1 cells during differentiation (Jung et al., 2018a). GPR120, also called free fatty acid receptor 4, is a free fatty acid receptor. Recently, DHA is found to promote adipogenesis by activating GPR120 in the cilia of preadipocytes. For the mechanism, GPR120 activation induced a rapid increase in ciliary cyclic AMP (cAMP) level, which in turn promoted adipogenesis by activating exchange factor directly activated by cAMP (EPAC) (Hilgendorf et al., 2019). Because GPR120 can be activated by PDX, this research implies the complicated effects of ω-3 PUFA-derived bioactive lipids on adipogenesis. In addition, more studies are needed to demonstrate whether ω-3 derived bioactive lipids can affect the lipid storage and release function of adipose tissue *in vivo*.

### Effect of ω-3 PUFA-Derived Oxylipins on Endocrine Function of Adipose Tissue

Adipose tissue, as an endocrine tissue, can affect other tissue functions by secreting cytokines. MaR1, 18-HEPE, 17-HDHA, RvD1, and PD1 could increase adiponectin level (Gonzalez-Periz et al., 2009; Hellmann et al., 2011; Rius et al., 2014; Martinez-Fernandez et al., 2017; Rodriguez-Echevarria et al., 2018). Adiponectin is an adipose-derived cytokine, one of the most abundant proteins in circulation (Wang et al., 2010). Because adiponectin is beneficial for diabetes, inflammation, and atherosclerosis (Achari and Jain, 2017), these bioactive lipids may affect metabolic disorders indirectly by promoting adiponectin secretion, which needs to be further explored.

### Effect of ω-3 PUFA-Derived Oxylipins on Brown and Beige Adipose Tissue

Brown and beige adipocytes, as heat-producing cells, are considered to counteract metabolic diseases, including obesity and type 2 diabetes. Leiria et al. (2019) found that the 12-LOX biosynthetic pathway was activated in brown adipose tissue under cold exposure, which promoted the generation and release of 12-HEPE. Then, 12-HEPE exerted a glucose-shuttling effect on tissues to support thermogenesis (Leiria et al., 2019).

GPR120 is highly expressed in brown adipose tissue and significantly upregulated in beige adipose tissue induced by cold exposure. It was further found to mediate ω-3 PUFA-induced thermogenic gene expression in beige adipocytes by upregulating fibroblast growth factor 21 expression (Quesada-Lopez et al., 2016). However, the role of ω-3 PUFA metabolites in white adipose tissue browning remains unknown. GPR120 can be activated by PDX, but whether these ω-3 PUFA-derived bioactive lipids could regulate this process is worth studying.

Besides the direct effects on adipose tissue, ω-3 PUFA metabolites are reported to indirectly regulate adipose tissue function. GPR18, the receptor for RvD2, is widely expressed in hypothalamus and was decreased in level by HFD feeding in mice. In addition, the production of hypothalamic RvD2 was decreased in HFD-fed mice. When obese mice were treated with intra-cerebroventricular injection of RvD2, visceral fat was reduced, and hypothalamic leptin resistance was reversed (Pascoal et al., 2017).

## Effect of ω-3 PUFA-Derived Oxylipins on Atherosclerosis

Atherosclerosis causes ischemic heart disease, strokes, and peripheral vascular disease (Kobiyama and Ley, 2018). Metabolic syndrome is responsible for the initial disease and disease progression (Varghese et al., 2018). Endothelial-cell dysfunction is the initial step of atherosclerosis. Plaque is chronically built up with the assistance of macrophages differentiated from monocytes, smooth muscle cells and multiple chemokines and growth factors (Gimbrone and Garcia-Cardena, 2016). The metabolites derived from EPA or DHA, including RvE1, RvD2, MaR1, 18-HEPE, and 17,18-EEQ, have shown positive effects on anti-atherosclerosis (Table 2).

Systematic plasma lipidomic research has identified 18-HEPE as a central molecule derived from EPA. 18-HEPE is an RvE1 precursor, and knockout of the RvE1 receptor ERV-1 enhanced atherosclerosis and promoted changes in plaque composition in ApoE–/– mice. The mechanism study showed that ERV-1/ChemR23–/– macrophages enhanced oxidized low-density lipoprotein uptake and decreased phagocytosis (Laguna-Fernandez et al., 2018). RvE1 can ameliorate atherosclerosis (Salic et al., 2016). In addition, 18-HEPE and 17,18-EEQ ameliorated endothelial-cell activation and monocyte adhesion by inhibiting the TNFα-induced NF-κB pathway (Liu et al., 2018).

In the ApoE–/– mouse aorta, RvD2 and MaR1 levels are correlated negatively with vulnerability plaque index, which is decreased by HFD treatment. In addition, RvD2 and MaR1 administration suppressed atheroprogression. The protective effects of RvD2 and MaR1 on atherosclerosis were mediated by preventing the macrophage inflammatory response (Viola et al., 2016). RvD1 was decreased in vulnerable regions as compared with stable regions in human carotid atherosclerotic plaques. Additionally, its level was decreased in advanced versus early atherosclerotic lesion in western diet-fed mice deficient in low-density-lipoprotein receptor (Fredman et al., 2016). These studies suggest that several metabolites of EPA and DHA are beneficial for atherosclerosis. However, more *in vivo* and mechanistic studies are needed to better understand their effects on atherosclerosis.

## Effect of n-3 DPA and Its Derivatives on Metabolic Disorders

n-3 DPA, an important ω-3 PUFA, is also a precursor of various docosanoids. Besides, it is an important intermediate in the conversion pathway of EPA and DHA (Figure 2; Drouin et al., 2019). n-3 DPA supplement significantly improved homeostasis model assessment of insulin resistance (HOMA-IR) in HFD fed mice, while DHA and EPA showed a minor effect (Guo et al., 2018). In human, n-3 DPA and its pro-resolving mediators have beneficial effects on cardiometabolic disease (Li et al., 2018). Moreover, It has been proved to be more potent than EPA in inducing the differentiation process in preadipocytes, and inhibits the pro-inflammatory signaling pathways (Murali et al., 2014). Although, it showed more beneficial effects on those metabolic disorders mentioned above than EPA and DHA, the functions of its metabolites are poorly studied.

n-3 DPA can be metabolized into PD_n__–__3__DPA_, RvD_n__–__3__DPA_, MaR_n__–__3__DPA_, hydroxylated derivatives from n-3 DPA, 13-serie Rvs etc. Several functional studies about n-3 DPA derivatives indicates their anti-inflammation function. A recent study found significant decreases in plasma RvD_n–__3__DPA_ concentrations in CVD patients and RvD_n–__3__DPA_ reduce leukocyte and platelet activation in peripheral blood from healthy volunteers as well as CVD patients. In addition, RvD5_n–__3__DPA_ reduced aortic lesions in western diet-fed ApoE–/– mice (Colas et al., 2018). PD_n–__3__DPA_ also found to play an important role in regulating macrophage resolution responses (Pistorius et al., 2018). PD1_n–__3__DPA_ and RvD5_n–__3__DPA_ were reported to decrease leukocyte–endothelial interaction and attenuate intestinal inflammation (Gobbetti et al., 2017). Although these n-3 DPA derivatives are identified as novel specialized proresolving lipid mediators, their effects on metabolic disorders, such as diabetes, NAFLD, obesity and atherosclerosis are still largely unknown.

## Effect of ALA and Its Derivatives on Metabolic Disorders

In addition to partially converted into EPA, n-3 DPA and DHA (with low conversion rate to DHA in human) (de Lorgeril and Salen, 2004; Stark et al., 2008, 2016), the effects of oxylipins derived from ALA by LOX and CYP have also gained attention. Recently, a clinical research showed that 9-hydroxy-octadecatrienoic acid (9-HOTRE) combined with 7,17dihydro-dipicolinic acid (7,17-DHDPA), 14,15-dihydroxy-5,8,11-eicosatrienoic acid (14,15-DIHETRE) and free adrenic acid is a biomarker to predict improvement in hepatic collagen content in NASH patients (Caussy et al., 2020). Besides, in obese rats, 9-HOTRE showed a negative correlation with mean glomerular volume (Caligiuri et al., 2013). 13-Oxo-9(Z),11(E),15(Z)-octadecatrienoic acid (13-oxo-OTA), a product from ALA catalyzed by LOX, was reported to promote glucose uptake in 3T3-L1 cells by activating PPARγ (Takahashi et al., 2015). Moreover, 13-(*S*)-hydroperoxyoctadecatrienoic acid [13-(S)-HPOTRE] and 13-(S)-hydroxyoctadecatrienoic acid [13-(S)-HOTRE] showed anti-inflammatory effects by inactivating NLRP3 inflammasome complex in macrophages, which indicates that they may play protective roles in metabolic disorders (Kumar et al., 2016). However, the studies about the effects of ALA derivatives on metabolic are limited, especially the *in vivo* study.

## Conclusion

Although we have fewer studies of the biofunctions of ω-3 PUFA-derived bioactive lipids than ARA metabolites, the former have been increasingly emphasized recently, especially for metabolic disorders (Table 2). Most of the functional studies focused on their anti-inflammatory effects. These metabolites can be more effective against inflammation than the precursors *per se*. Because PUFAs are vulnerable to lipid peroxidation, ω-3 PUFA supplement can lead to increased lipid peroxidation products, which may limits their clinical applications (Zaloga, 2021). It is important to increase their anti-inflammatory efficiency and decrease the dosage. Therefore, studying the function ω-3 PUFA metabolites may help us to find novel lipid mediators to treat metabolic disorders better than dietary supplement of EPA and DHA. Moreover, the anti-inflammatory efficiency of these metabolites should be further compared to provide more information for the future clinical applications.

In addition, several studies revealed the direct effects of ω-3 PUFA-derived oxylipins on pancreatic β cells, hepatocytes, adipocytes, skeletal muscle cells and endothelial cells. These bioactive lipids may have potential effects other than anti-inflammatory effects, which needs more exploration. Metabolites derived from ω-3 PUFAs are numerous, with attention to RvEs, RvDs, and PDs. Other metabolites such as EEQs, EDPs, HEPEs, and n-3 DPA derivatives need more mechanistic studies. In addition, the explorations of the biofunctions of ω-3 PUFA-derived bioactive metabolites, including their effects on cellular function, tissue micro-environment and interactions among metabolic tissues, are important for understanding their roles in energy metabolic disorders and related diseases. We also need more studies to identify their receptors and elucidate the downstream signaling pathway, which may provide potential therapeutic strategies for metabolic disorders.

In animal studies, the age, sex, and background of animals are well controlled. However, plasma and tissue levels of EPA and DHA and their metabolites in human can be altered by age, sex and disease status (Calder, 2020a), which indicates the complexity of clinical application of EPA and DHA. The genetic variants in the specialized pro-resolving mediator response also need to be considered when exploring the therapeutic effects of EPA and DHA clinically. Thus, the individualized treatment regimens of clinical applications of ω-3 PUFAs may achieve better effects on metabolic disorders. Moreover, according to a recent clinical trial, high-dose ω-3 PUFA supplement failed to increased RvD1 levels in diabetic patients, indicating the importance to study the disturbance of ω-3 PUFA metabolism in some disease status.

## Author Contributions

JD and YS contributed to the drafting, figure composition, subsequent edits, and final composition of the manuscript. XZ provided the comments and corrections. CW contributed to the concept and design, drafting of the manuscript, and guarantor of the manuscript. All authors have read and approved the final manuscript.

## Conflict of Interest

The authors declare that the research was conducted in the absence of any commercial or financial relationships that could be construed as a potential conflict of interest.

## Abbreviations

ALA: α-linolenic acid
ARA: arachidonic acid
AMPK: AMP-activated protein kinase
BLT: leukotriene B4 receptor
cAMP: cyclic AMP
CCL: C-C motif chemokine ligand
COX: cyclooxygenase
CYP: cytochrome P450
DHA: docosahexaenoic acid
DHEA: docosahexaenoyl ethanolamine
DiHDPA: dihydroxydocosapentaenoic acid
DiHETE: dihydroxyeicosatetraenoic acid
DPA: docosapentaenoic acid
EDP: epoxydocosapentaenoic acid
EEQ: epoxyeicosatetraenoic acid
EPA: eicosapentaenoic acid
EPEA: *N*-eicosapentaenoyl ethanolamine
ERV-1: Resolvin E1 Receptor
GPCR: G protein-coupled receptors
HDoHE: hydroxydocosahexaenoic acid
HEPE: hydroxyeicosapentaenoic acid
HFD: high-fat-diet
IFN- γ: interferon γ
IL: interleukin
LGR6: leucine-rich repeat containing G protein-coupled receptor 6
LOX: lipoxygenase
LXA4: lipoxin A4
MaR: maresin
NAFLD: non-alcoholic fatty liver disease
NASH: non-alcoholic steatohepatitis
PD: Protectins
PDX: Protectin DX
PPAR: peroxisome proliferator-activated receptor
PUFA: polyunsaturated fatty acid
RvD: D-series resolvin
RvE: E-series resolvin
sEH: soluble epoxide hydrolase
TNF- α: tumor necrosis factor α
7,17-DHDPA: 7,17dihydro-dipicolinic acid
9-HOTRE: 9-hydroxy-octadecatrienoic acid
13-(S)-HOTRE: 13-(*S*)-hydroxyoctadecatrienoic acid
13-(S)-HPOTRE: 13-(*S*)-hydroperoxyoctadecatrienoic acid
13-oxo-OTA: 13-Oxo-9(Z),11(E),15(Z)-octadecatrienoic acid
14,15-DIHETRE: 14,15-dihydroxy-5,8,11-eicosatrienoic acid.
